# Corneal safety assessment of germicidal far UV-C radiation

**DOI:** 10.1038/s41598-025-09241-2

**Published:** 2025-07-05

**Authors:** Daniela F. Zamudio Díaz, Patricia Hülse, Johannes Schleusener, Anja A. Kühl, Anna Lena Klein, Loris Busch, Lalita Roscetti, Martin Guttmann, Sascha Rohn, Thomas A. Fuchsluger, Martina C. Meinke

**Affiliations:** 1 Institute of Food Technology and Food Chemistry, Faculty III Process Sciences, Technische Universität Berlin, Gustav-Meyer-Allee 25, 13355 Berlin, Germany; 2 Center of Experimental and Applied Cutaneous Physiology, Department of Dermatology, Venereology and Allergology, Charité - Universitätsmedizin Berlin, Corporate Member of Freie Universität Berlin and Humboldt-Universität zu Berlin, Charitéplatz 1, 10117 Berlin, Germany; 3 Klinik und Poliklinik für Augenheilkunde, Universitätsmedizin Rostock, Doberaner Straße 140, 18057 Rostock, Germany; 4 iPATH.Berlin, Charité – Universitätsmedizin Berlin, Corporate Member of Freie Universität Berlin and Humboldt-Universität zu Berlin, Campus Benjamin Franklin, Hindenburgdamm 30, 12200 Berlin, Germany; 5 Ferdinand-Braun-Institut (FBH), Gustav-Kirchhoff-Str. 4, 12489 Berlin, Germany

**Keywords:** Cornea, Far UV-C, DNA damage, Optical properties, Penetration depth, DNA damage response, Computational biophysics, Lasers, LEDs and light sources, Risk factors, Translational research

## Abstract

Far UV-C radiation (200–240 nm) is a promising alternative to conventional UV-C for disinfection in occupied spaces, offering strong germicidal efficacy with reduced skin risk. However, its ocular safety remains unclear, as most studies relied only on non-human corneal models with physiological differences. This study investigated UV-induced DNA damage in the epithelium, stroma, and endothelium of ex vivo human corneas and porcine corneas, and reconstructed human cornea epithelium (RHCE) using immunohistochemistry. Samples were exposed to 222 nm, 233 nm, 254 nm, and broadband UV-B (280–400 nm) radiation in the presence of real human tears. Compared to human corneas (26 μm mean epithelium thickness), porcine corneas (110 μm) and RHCE (79 μm), showed reduced UV penetration. In human corneas with a thin epithelium, far UV-C exposure led to epithelial and anterior stromal damage, underscoring the epithelium’s protective function. Optical properties using porcine corneas confirmed the immunohistological findings, validating wavelength-dependent penetration depths. Simulations suggest that in intact human corneas, damage-relevant intensity of 222 nm light reaches the middle of the epithelium, while for 233 nm, it reaches the basal layer. These findings support the relative safety of far UV-C, especially 222 nm, for intact corneas. However, potential DNA damage accumulation after repeated exposures underscores the need for further research on long-term ocular effects.

**Supplementary Information:**

The online version contains supplementary material available at 10.1038/s41598-025-09241-2.

## Introduction

The COVID-19 pandemic forced the urgent need for innovative strategies to mitigate airborne microorganism transmission and reduce bacterial loads indoor such as doctors waiting rooms or infection ward in the hospital^1^. UV-C radiation, typically at 254 nm, is known for its strong germicidal properties, disrupting microbial DNA and impeding its replication without fostering antibiotic resistance^2^. However, its risks to human cells, including DNA lesions in skin and corneal cells, are well documented^3–5^. These include the prevalent DNA lesions cyclobutane pyrimidine dimers (CPD) and pyrimidine-pyrimidone (6 –4) photoproduct (6-4PP)^6^. The irradiation with 254 nm has been associated with cutaneous carcinogenesis and an array of ocular pathologies, ranging from photokeratosis to ocular neoplasms^7^.

Far UV-C radiation (200–240 nm) has received increasing attention due to its germicidal properties with minimal risk to human cells^8^. Unlike conventional UV-C radiation, far UV-C radiation effectively eradicates pathogens, including pathogenic viruses and yeast^9,10^while sparing mammalian cells due to pronounced absorption and scattering effects within the superficial skin layers^11^. Thus, making it an attractive tool for disinfection in environments where humans are present^1^.

The ocular surface is a complex anatomical structure that includes the eyelid margin, the tear film, the outer layer of the cornea, and the conjunctiva^12^. The cornea serves as the primary barrier against environmental factors while enabling vision through refraction^13^.

The cornea consists of five layers: epithelium, Bowman’s membrane, stroma, Descemet’s membrane, and endothelium^13^. Comprising 10% of the corneal thickness (five to seven cell layers), the corneal epithelium, a stratified, squamous, nonkeratinized layer, safeguards the cornea from UV rays, pollutants, and injury, while facilitating cell renewal and maintaining barrier functions essential for corneal integrity^14,15^. Beneath it, the stroma, about 500 μm thick, provides structure and transparency through organized collagen fibers^16^. The corneal endothelium, a monolayer of cells on the posterior corneal surface, regulates fluid transport, crucial for maintaining optical clarity and normal corneal thickness^17^. The anterior parts of the eye, particularly the cornea and the lens, absorb over 99% of natural UV radiation, with the stroma absorbing UV-B (< 300 nm) and the lens absorbing UV-A (< 370 nm). The effects of UV radiation become more severe when endothelial cells are affected, because of their poor ability to proliferate, underscoring the critical importance of preserving the integrity of these cells for ocular health^13,18^.

Several studies confirm the cutaneous safety of far UV-C radiation^1,11,19,20^but its effects on the human cornea remain less explored. Studies indicate that 222 nm radiation is tolerable for the eye, with corneal integrity maintained at exposure doses up to 600 mJ/cm^2^^21^. Furthermore, 222 nm irradiation only effectively penetrates the corneal epithelium at doses above 1,500 mJ/cm2^22^. No evidence of CPD formation has been found in corneal limbal stem cells at 600 mJ/cm^2^ with 222 nm in rat and pig models^21^with normal corneal limbal stem cell function and normal epithelial turnover^22^. Although far UV-C radiation appears to be safe for the human cornea, most studies have been conducted in different ocular models, such as the rat and porcine cornea, limiting clinical translation. Traditional models, such as animal corneas and cell culture models, differ physiologically from human corneas, particularly in tissue depth^23^.

As far UV-C radiation emerges as a promising disinfection tool in human environments, understanding its potential ocular risks is critical. Consequently, this study aimed at characterizing UV-induced DNA damage in reconstructed human cornea epithelium (RHCE), ex vivo human cornea samples, and ex vivo porcine cornea samples, with a specific focus on far UV-C irradiation. To complement the biological assessment, corneal optical properties and wavelength-dependent penetration depths were also simulated. By integrating experimental and computational approaches, this work contributes important insights into corneal vulnerability to UV exposure and supports informed decisions about the safe use of far UV-C in human environments.

## Results

### Reconstructed human cornea epithelium (RHCE)

**Fig. 1:**
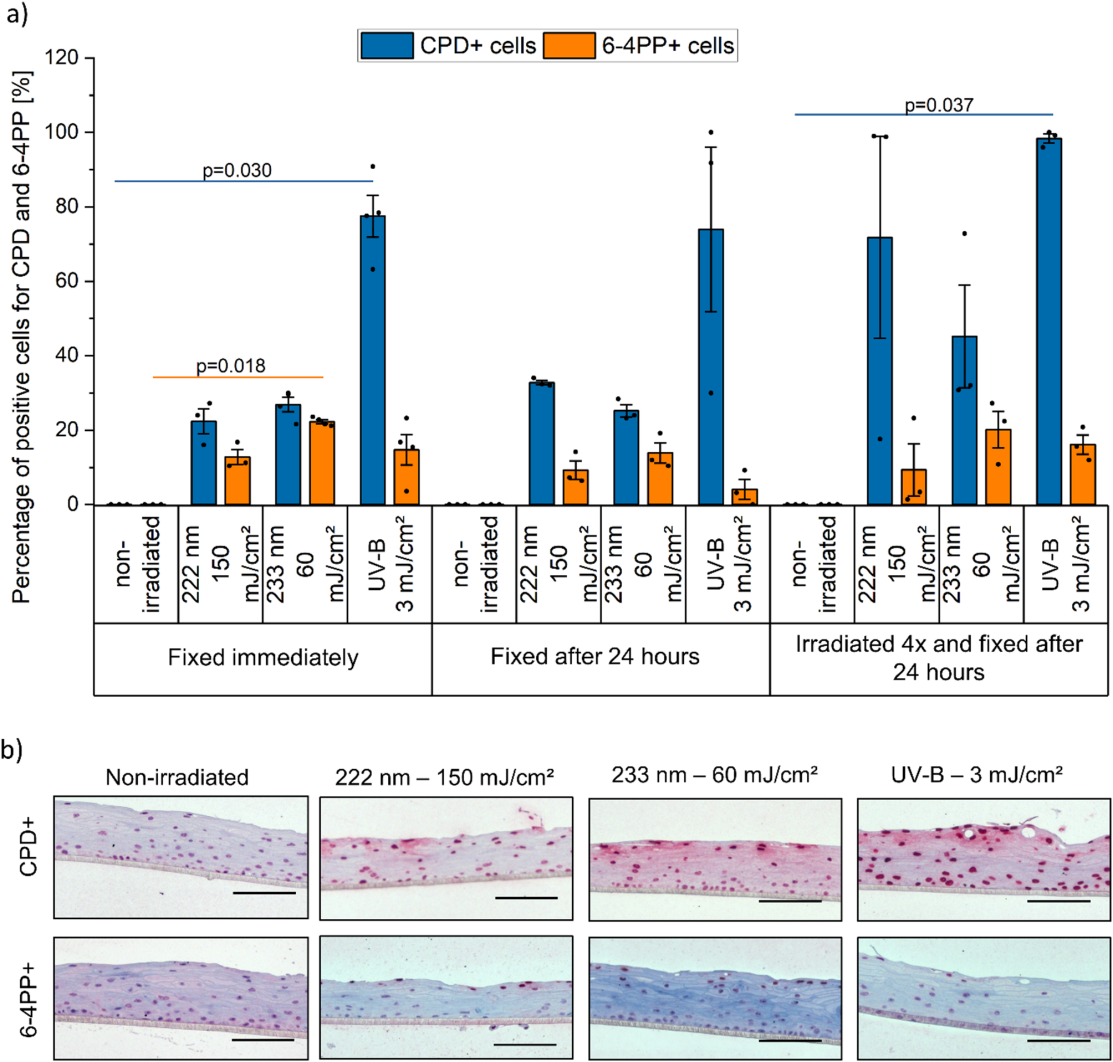
UV-induced DNA damage in the epithelium of reconstructed human cornea (RHCE). (**a**) DNA damage, as percentage of cells with cyclobutane pyrimidine dimers (CPD, blue) and pyrimidine-pyrimidone (6 − 4) photoproduct (6-4PP, orange) formation, determined by immunohistochemistry, fixed immediately and 24 h after single or multiple UV irradiation (222 nm–150 mJ/cm^2^, 233 nm–60 mJ/cm^2^, and broadband UV-B (280–400 nm)﻿–3 mJ/cm^2^). Different tissue sections were stained separately for each lesion type using lesion-specific antibodies. Pairwise group differences were assessed using one-way ANOVA followed by Bonferroni-corrected post hoc tests. Far UV-C irradiation induces superficial damage in epithelial cells, with no significant repair observed. Following multiple irradiations, CPD damage accumulates. Data represents mean ± SEM (*n* = 3–4). (**b**) Representative images of immunohistochemical detection of CPD and 6-4PP positive cells in the epithelium of RHCE. Positive cells are stained in dark red. Scale bar: 100 μm.

First, reconstructed human corneas (*n* = 4) were used to evaluate UV-induced DNA damage after a single exposure, 24 h repair, and multiple exposures with 222 nm, 233 nm, and UV-B (hereafter referring to a broadband UV-B source spanning 280–400 nm with a peak emission at 311 nm) (Table 1; Fig. 8). Immediate tissue fixation revealed comparable and superficial CPD-positive (CPD+) cell numbers for both far UV-C wavelengths, while UV-B exposure resulted in a threefold increase (> 75%). An increase in 6-4PP+ cells was observed for all evaluated wavelengths; however, due to variability in the results, the increase was only statistically significant following 233 nm irradiation (Fig. 1). The mean epithelial thickness of all samples after exposure, based on immunohistological images, was 79 ± 2 μm.

Although a slight repair of 6-4PP+ cells was observed in the corneal tissue after 24 h across all irradiated groups, no significant repair of CPDs (*p* = 1.000) was observed.

Four repeated irradiations with two hour breaks in between, followed by a 24 h re-cultivation phase, led to the highest CPD levels, while no clear trend was observed for 6-4PP. CPD formation did not increase fourfold compared to a single dose in any group. At 222 nm and 233 nm, CPD levels approximately doubled, whereas cumulative UV-B exposure led to saturation, with a significant increase of about one-third in CPD+ cells. Variability in the results following multiple irradiations likely due to the effects of re-cultivation and repeated irradiation, which may compromise model integrity.

### Human cornea samples

Due to the absence of all corneal layers, increased epithelial thickness in RHCE, and limited damage repair after 24 h, irradiation experiments were conducted on human corneal samples in the presence of human tears to better simulate in vivo conditions. Damage was evaluated in the different layers of the cornea, including epithelium, stroma, and endothelium, both directly and 24 h after irradiation (Table 1). The mean epithelial thickness of all samples after exposure, based on immunohistological images, was 26 ± 2 μm. The depth of damage was determined based on the appearance of CPD + cells, measured at five points on histological images (Supplementary Fig. 1).

#### Immunohistochemical assessment of DNA damage in epithelium

**Fig. 2:**
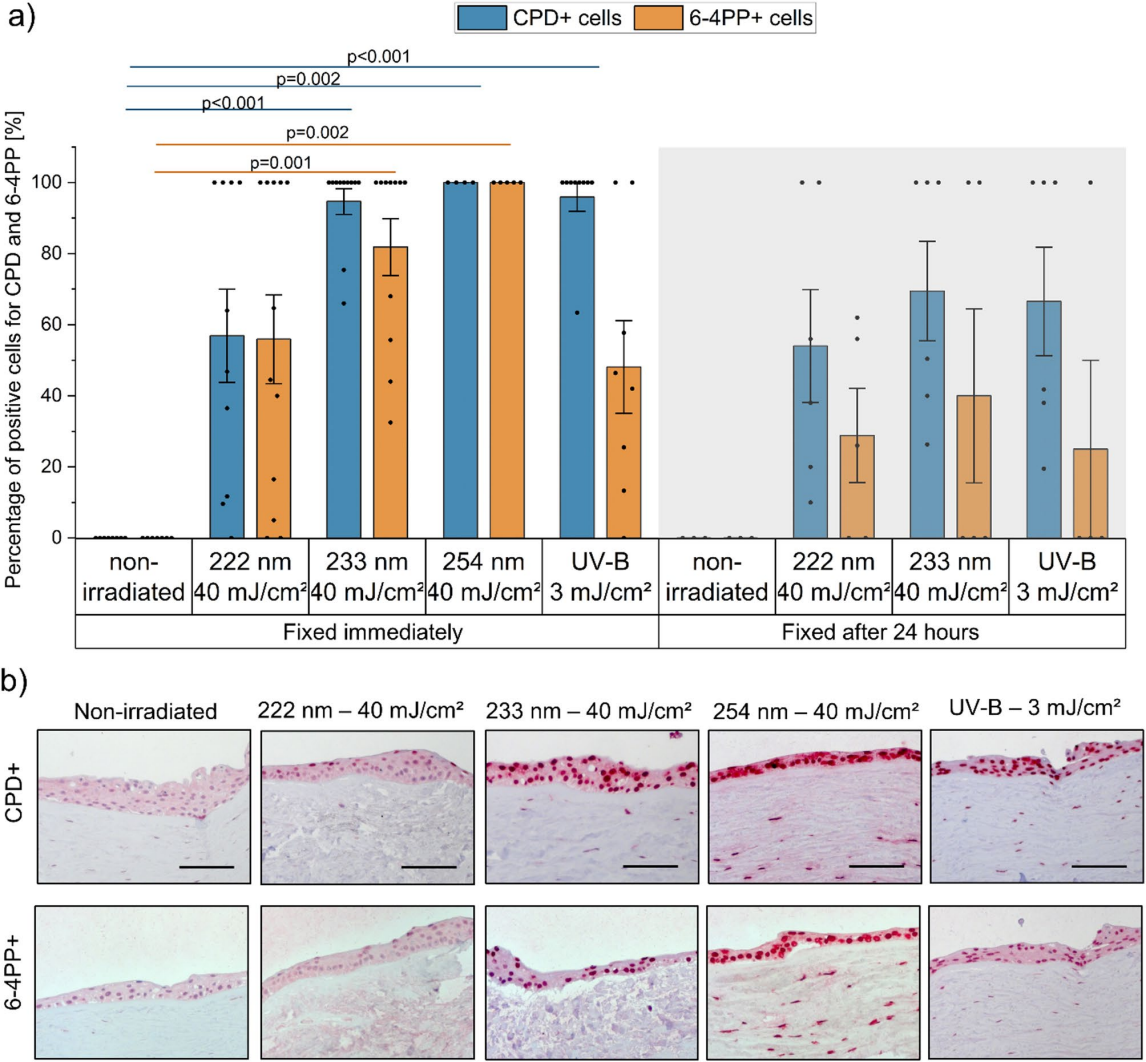
UV-induced DNA damage in the epithelium of human cornea. (**a**) DNA damage, as percentage of cells with cyclobutane pyrimidine dimers (CPD, blue) and pyrimidine-pyrimidone (6 − 4) photoproduct (6-4PP, orange) formation, determined by immunohistochemistry, fixed immediately and 24 h after UV irradiation. (222 nm–40 mJ/cm^2^, 233 nm–40 mJ/cm^2^, 254 nm–40 mJ/cm^2^, and broadband UV-B (280–400 nm)﻿–3 mJ/cm^2^). Different tissue sections were stained separately for each lesion type using lesion-specific antibodies. Pairwise group differences were assessed using one-way ANOVA followed by Bonferroni-corrected post hoc tests. Almost all epithelial cells showed DNA damage after 233 nm, 254 nm and UV-B irradiation and more than 50% after 222 nm irradiation. Considerable repair was observed 24 h after exposure. Data represents mean ± SEM (*n* = 4–11). (**b**) Representative images of immunohistochemical detection of CPD and 6-4PP positive cells in the epithelium of human cornea samples. Positive cells are stained in dark red. Scale bar: 100 μm.

Immunohistochemical analysis of DNA damage (CPD+ and 6-4PP+ cells) and on repair (damage 24 h after exposure) revealed wavelength-dependent variations in penetration depth. CPD damage immediately after irradiation with 233 nm, 254 nm and UV-B was significantly higher compared to non-irradiated samples. UV-B, far UV-C at 233 nm, and 254 nm irradiation induced DNA damage in nearly all epithelial cells (approximately 100%). Notably, there was no significant difference between far UV-C irradiation at 233 nm and conventional UV-C irradiation at 254 nm (*p* = 1.000) (Fig. 2). In contrast, irradiation at 222 nm resulted in only 60 ± 12% CPD+ cells observed at a depth of approximately 27 ± 4 μm.

Although not significantly different, a reduction in CPD+ cells was observed 24 h after exposure for all wavelengths (*p* = 1.000). Repair was quantified by comparing the mean percentage of CPD+ cells immediately after exposure and 24 h post-exposure. Based on this, approximately 30% CPD lesions were repaired 24 h after exposure to UV-B radiation, 27% for 233 nm and 11% for 222 nm. In all cases, CPD + cells remained below 65%.

In general, 6-4PP formation was less prevalent than the CPD formation. In corneas exposed to 254 nm, all cells exhibited 6-4PP formation, followed by corneas exposed to 233 nm (80 ± 8% 6-4PP). Both irradiated groups (254 nm and 233 nm) showed significant differences compared to the non-irradiated samples. In contrast, samples irradiated with 222 nm or UV-B showed approximately 50 ± 12% 6-4PP + cells and were not significantly different from the non-irradiated samples (*p* = 0.067 and *p* = 0.223, respectively). Although not statistically significant (*p* = 1.00), repair after 233 nm irradiation was considerable, with less than 40 ± 22% of epithelial cells showing 6-4PP at 24 h.

#### Immunohistochemical assessment of DNA damage in stroma

To assess the UV-induced damage to the stroma, it was divided into anterior stroma (upper, subepithelial) and posterior stroma (lower, supraendothelial) (Supplementary Fig. 2). Due to its thickness, the stroma showed less DNA damage than the epithelium.

**Fig. 3:**
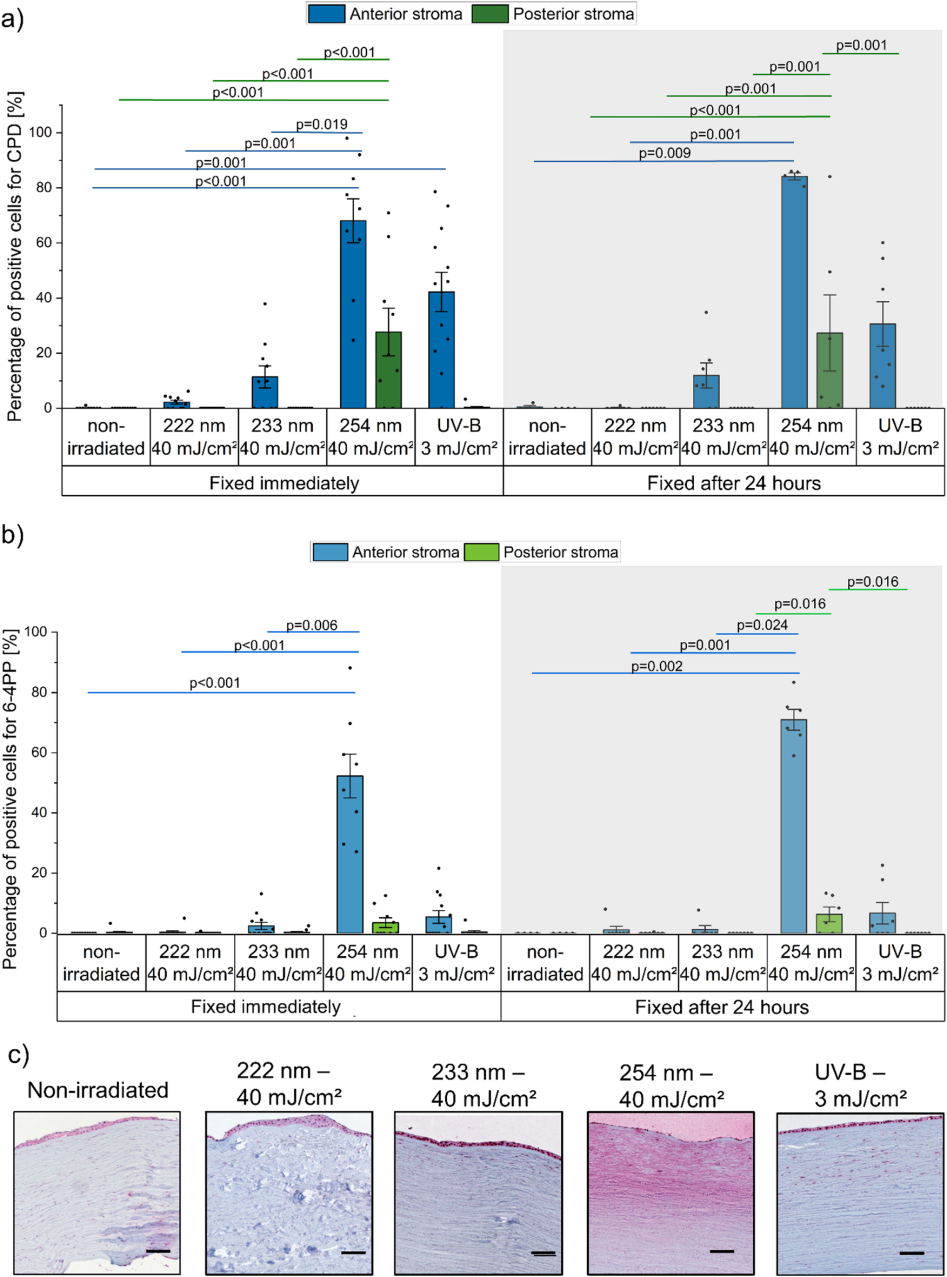
UV-induced DNA damage in the anterior (blue) and posterior stroma (green) of human cornea. (**a**) DNA damage, as percentage of cells with cyclobutane pyrimidine dimers (CPD) and (**b**) pyrimidine-pyrimidone (6 − 4) photoproduct (6-4PP) formation, determined by immunohistochemistry, fixed immediately and 24 h after UV irradiation (222 nm–40 mJ/cm^2^, 233 nm–40 mJ/cm^2^, 254 nm–40 mJ/cm^2^, and broadband UV-B (280–400 nm)–3 mJ/cm^2^). Different tissue sections were stained separately for each lesion type using lesion-specific antibodies. Pairwise group differences were assessed using one-way ANOVA followed by Bonferroni-corrected post hoc tests. Minimal and superficial damage was detected in the anterior stroma after irradiation with 233 nm, and almost no damage was observed following irradiation with 222 nm. UV-B irradiation induced damage in the anterior stroma, whereas 254 nm penetrated deeper, reaching the posterior stroma. Lower repair was observed. Data represents mean ± SEM (*n* = 5–14). (**c**) Representative images of immunohistochemical detection of CPD-positive cells in the stroma of human cornea samples. Positive cells are stained in dark red. Scale bar: 100 μm.

The anterior stroma exhibited significantly greater damage in corneas irradiated with 254 nm at 40 mJ/cm^2^ and with UV-B at 3 mJ/cm^2^ compared to those exposed to 233 nm or 222 nm at 40 mJ/cm^2^. For the UV-B radiation a CPD damage depth of 166 ± 25 μm was stablished (Supplementary Fig. 1). Irradiation at 233 nm resulted in 14 ± 5% CPD+ cells, with a damage depth of approximately 53 ± 8 μm. In contrast, 222 nm irradiation induced less than 5 ± 1% CPD+ cells (Fig. 3).

In the posterior stroma, no CPD+ cells were detected after exposure to both far UV-C sources and the UV-B lamp. In contrast, the exposure to 254 nm still managed to penetrate deeply (21 ± 8% CPD+ cells), showing significant differences with the non-irradiated group. As shown in Fig. 3, irradiation at 40 mJ/cm² with 254 nm, caused some visible tissue disruption; however, quantification of DNA damage remained feasible due to the presence of intact nuclei and the nuclear specificity of the staining. Further studies will be required to assess tissue integrity following such exposure.

While not statistically significant (*p* = 1.000), there was a tendency for stromal damage repair at 24 h post-irradiation, which was less pronounced than in the epithelium. For the 233 nm, a damage repair of 14% (12 ± 5% CPD+ cells 24 h after exposure, *p* = 1.000) and for UV-B, a repair of 26% was observed (*p* = 1.000). No DNA damage was detected 24 h after exposure to 222 nm. Contrary to the previous cases, a slight increase in CPD+ cells was observed 24 h after exposure to 254 nm (Fig. 3).

Similar to the epithelium, 6-4PP formation was markedly lower than CPD. Only a predominant 6-4PP formation was evident after 254 nm irradiation in the anterior stroma, showing significant differences with the non-irradiated, 222 nm and 233 nm groups. Again, a slight increase in 6-4PP + cells was observed 24 h after 254 nm exposure.

#### Immunohistochemical assessment of DNA damage in the endothelium

**Fig. 4:**
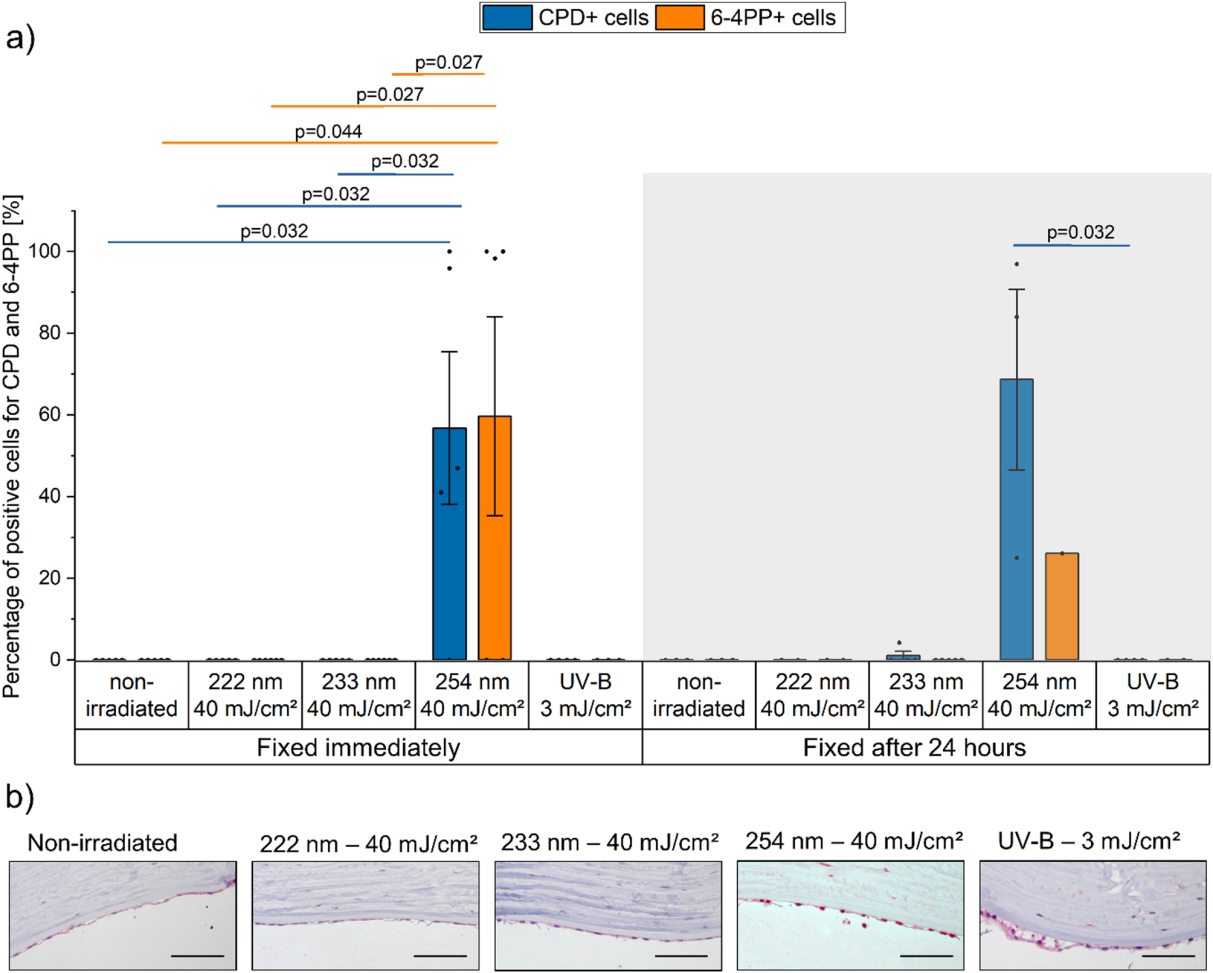
UV-induced DNA damage in the endothelium of human cornea. (**a**) DNA damage, as percentage of cells with cyclobutane pyrimidine dimers (CPD, blue) and pyrimidine-pyrimidone (6 − 4) photoproduct (6-4PP, orange) formation, determined by immunohistochemistry, fixed immediately and 24 h after UV irradiation. (222 nm–40 mJ/cm^2^, 233 nm–40 mJ/cm^2^, 254 nm–40 mJ/cm^2^, and broadband UV-B (280–400 nm)–3 mJ/cm^2^). Different tissue sections were stained separately for each lesion type using lesion-specific antibodies. Pairwise group differences were assessed using one-way ANOVA followed by Bonferroni-corrected post hoc tests. Only irradiation at 254 nm induced endothelial damage. Data represents mean ± SEM (*n* = 2–8). (**b**) Representative images of immunohistochemical detection of CPD-positive cells in the endothelium of human cornea samples. Positive cells are stained in dark red. Scale bar: 100 μm.

In the deepest layer of the cornea, the endothelium, cells remained unstained for CPD or 6-4PP lesions after the most irradiations, including 222 nm at 40 mJ/cm^2^, 233 nm at 40 mJ/cm^2^, and UV-B at 3 mJ/cm^2^. However, similar to the stroma, 254 nm at 40 mJ/cm^2^ caused significant CPD+ and 6-4PP+ cells, differing from the non-irradiated, 222 nm, and 233 nm groups. After 254 nm exposure, repair of 6-4PP lesions was observed after 24 h, but CPD lesions increased from 57 ± 19% to 69 ± 22% (Fig. 4).

### Porcine cornea samples

Due to the thin epithelium in human cornea samples (also referred to in this study as non-intact epithelium), the penetration results were found to be compromised (see Supplementary Fig. 1 for the depth of DNA damage in the human cornea). To address this issue, porcine corneal samples (*n* = 2) were included as a third model in the corneal tolerance studies. These samples were irradiated following the same protocol as the human corneas, including the application of human tears during irradiation to simulate tear film conditions and prevent desiccation. This was an exploratory experiment designed to qualitatively assess UV penetration behavior in intact tissue and was not a primary focus of the study. Due to the small sample size, no statistical testing was conducted.

A DNA damage assessment was conducted on the three corneal layers: the epithelium, stroma, and endothelium. Nevertheless, damage assessment in the endothelium was not feasible due to the non-intact structural integrity of the tissue in all the samples. In several cases, the epithelium was either absent in the histological section or completely or partially detached from the stroma.

**Fig. 5:**
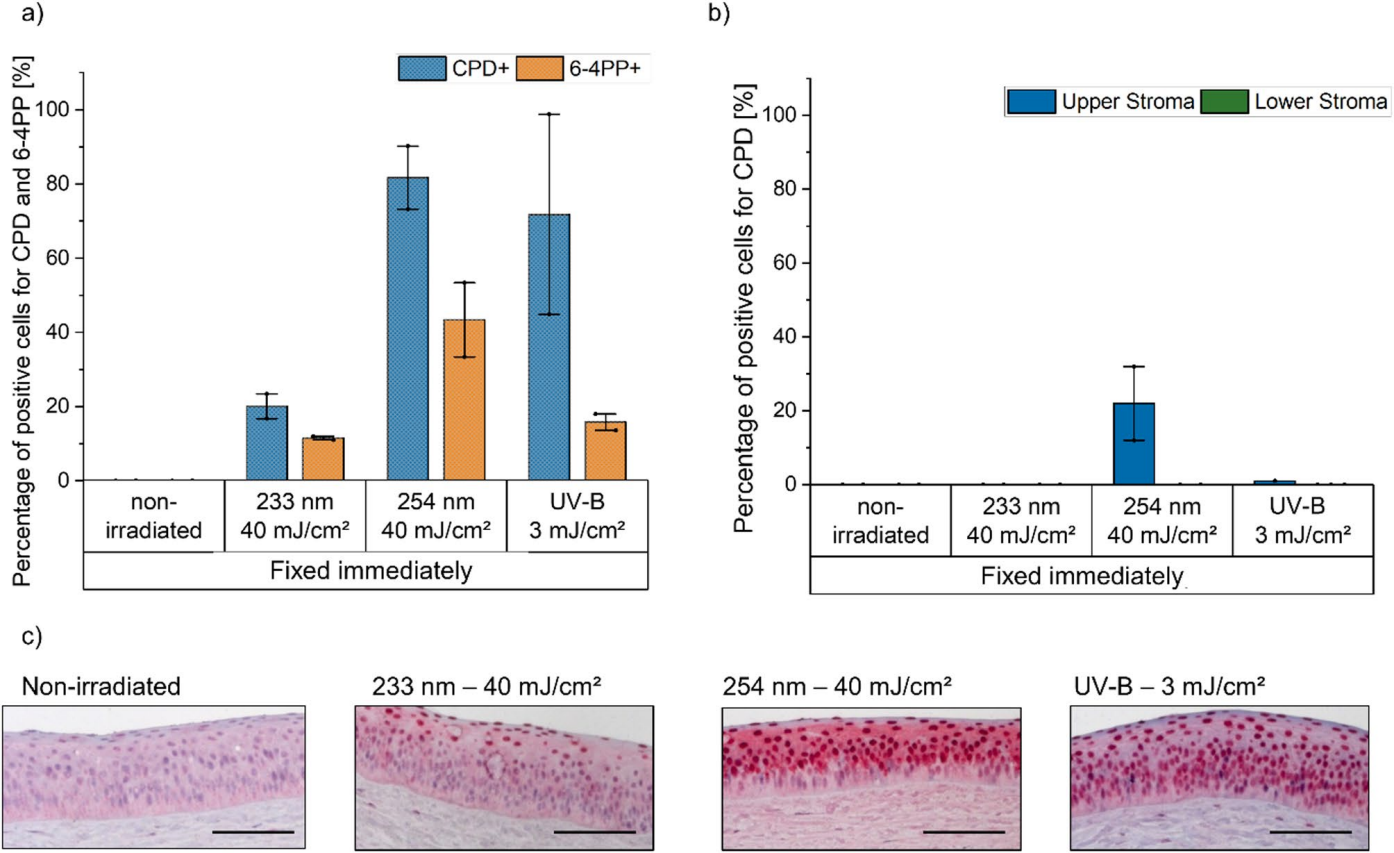
UV-induced DNA damage in the (**a**) epithelium, (**b**) anterior and posterior stroma of porcine cornea. DNA damage, as percentage of cells with cyclobutane pyrimidine dimers (CPD, blue and green) and pyrimidine-pyrimidone (6 − 4) photoproduct (6-4PP, orange) formation, determined by immunohistochemistry, fixed immediately and 24 h after UV irradiation (233 nm–40 mJ/cm^2^, 254 nm–40 mJ/cm^2^ and broadband UV-B (280–400 nm)–3 mJ/cm^2^). Irradiation at 233 nm induces superficial damage to the epithelium, UV-B causes more extensive damage throughout the epithelium, and 254 nm penetrates into the anterior stroma, inducing the highest level of damage. Data represents mean ± SEM (*n* = 2). (**c**) Representative images of immunohistochemical detection of CPD-positive cells in the epithelium of porcine cornea samples. Positive cells are stained in dark red. Scale bar: 100 μm.

Similar to the wavelength-dependent behavior observed in human corneal samples and RHCE, porcine corneal samples demonstrated pronounced damage to the epithelium following exposure to 254 nm (82 ± 8% CPD+ cells) and UV-B (72 ± 27% CPD+ cells), with 233 nm exhibiting a comparatively lesser impact (Fig. 5). Given the thickness of the epithelium in porcine samples (110 ± 2 μm), the depth and levels of damage were less than those observed in human corneal samples but comparable to those seen in RHCE (epithelium thickness 79 μm ± 2 μm) (Supplementary Fig. 1 for the depth of DNA damage in the porcine cornea). Consequently, irradiation with 233 nm at 40 mJ/cm^2^ resulted in 20 ± 3% CPD+ cells with a mean maximum penetration of 28 ± 5 μm. After irradiation with 254 nm CPD lesions were detected in the anterior stroma, with a mean penetration 71 ± 2 μm. In the posterior stroma, none of the irradiations were able to induce DNA lesions. 6-4PP+ cells were only observed in the epithelium, with 12 ± 0% observed after 233 nm, 16 ± 2% after UV-B, and 22 ± 10% of 6-4PP+ cells after 254 nm.

### Absorption and scattering coefficients in epithelium and stroma

**Fig. 6:**
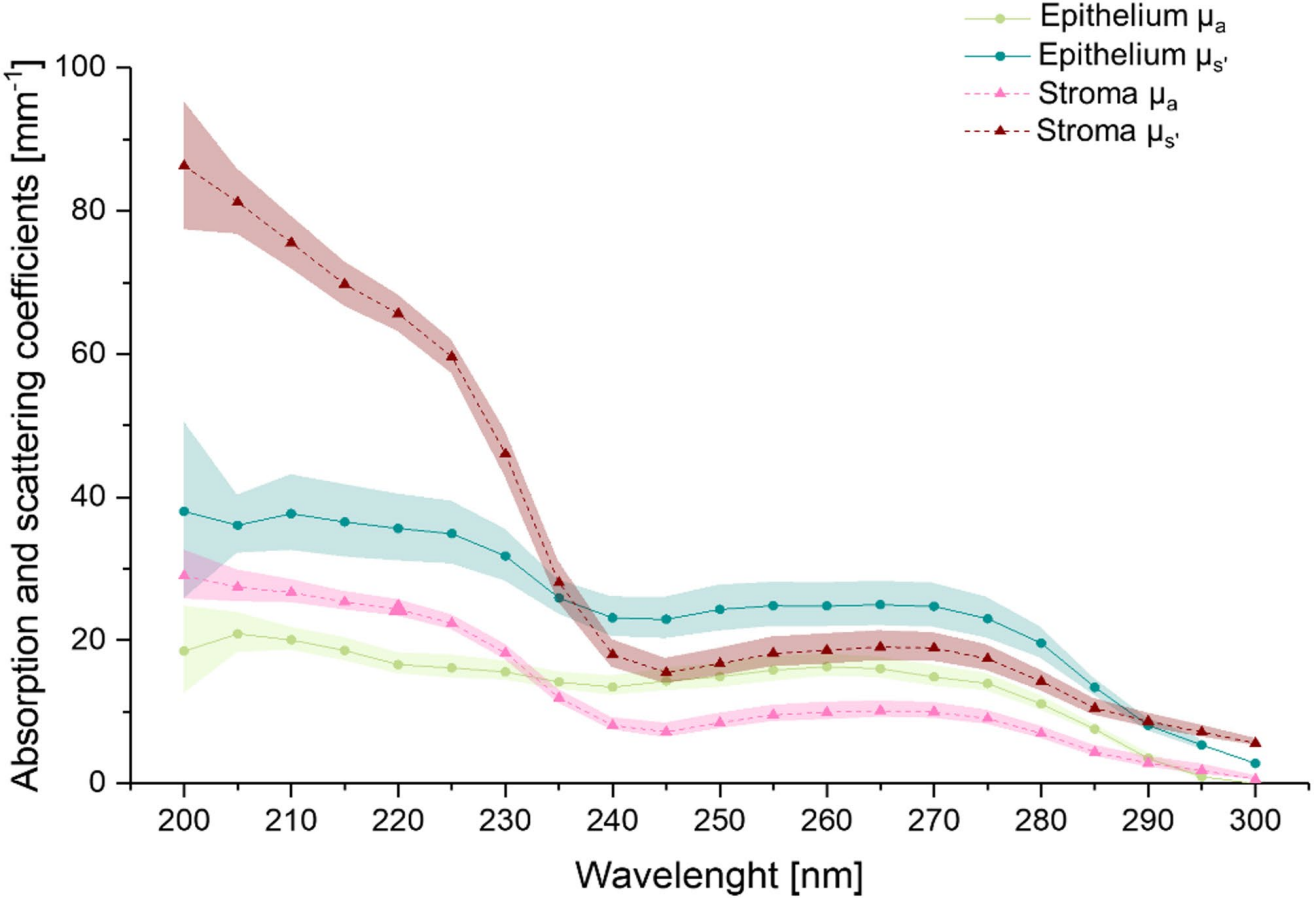
Optical properties of the porcine cornea obtained with the inverse Monte Carlo simulation (iMCS) for the wavelengths 200 nm to 300 nm for a spectral bandwidth of 5 nm. The simulated absorption coefficient µ_a_ and scattering coefficient µ_s_’ of the epithelium and stroma, from 40 μm thin sections. µ_s_’ is higher than µ_a_ in both layers. Below 240 nm, both coefficients increase, with the stroma showing higher values than the epithelium. *n* = 3 porcine cornea samples, *n* = 18 thin sections for stroma and *n* = 5 thin sections for epithelium. All data are expressed as mean ± SEM.

Immunohistological analysis of the three corneal models showed a clear correlation between UV-induced DNA damage and epithelial thickness. To better estimate the light penetration depth in an intact human cornea with normal epithelial thickness, the optical properties of the porcine cornea, specifically absorption (µ_a_) and effective scattering coefficients (µ_s_’), were determined (Fig. 6). A total of *n* = 3 porcine corneas were analyzed, with *n* = 4–6 thin sections per cornea for the stroma and *n* = 1–3 thin sections for the epithelium.

In both layers, the effective scattering coefficient µ_s_’ is higher than the absorption coefficient µ_a_. In the epithelium and stroma, the coefficients remain relatively constant between 240 and 280 nm, while an evident increase occurs below 240 nm. A variation in behavior at 240 nm is observed when comparing the two layers. Between 240 and 300 nm, the epithelium exhibits higher µ_a_ and µ_s_’; however, for wavelengths shorter than 240 nm, the stroma displays higher µ_a_ and µ_s_’.

### Penetration depth

**Fig. 7:**
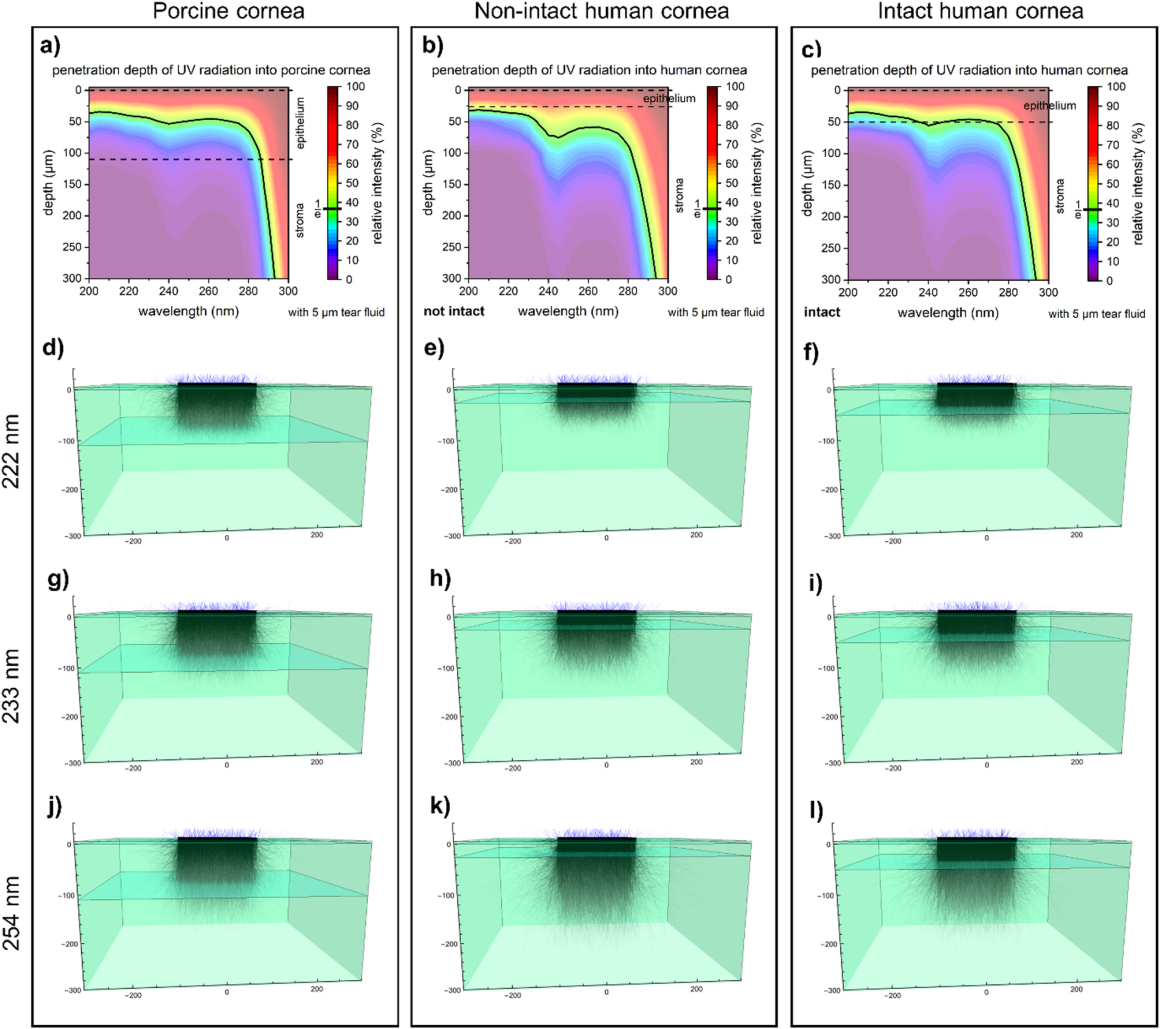
Simulation of penetration depth in porcine cornea (**a**), human cornea with non-intact (thin) epithelium (**b**), human cornea with intact epithelium (**c**) in the wavelength range from 200 to 300 nm using forward Monte Carlo simulation. The simulations in the human cornea were based on the optical properties of the porcine cornea. Light penetration depends on wavelength and epithelium integrity. In human corneas, thinning of the epithelium increases penetration. Furthermore, simplified 3D models with light propagation of 222 nm (**d**,** e**,** f**), 233 nm (**g**,** h**, **i**), 254 nm (**j**,** k**, **l**) show that penetration depth increases with wavelength: 254 nm > 233 nm > 222 nm. The authors refer the reader to the online version of this article for a color representation.

Based on the absorption and reflection coefficients measured for the porcine corneas, the penetration depth of light with wavelengths between 200 and 300 nm was simulated using a forward Monte Carlo simulation for the porcine cornea and for the human cornea without (referred to as thin epithelium) and with intact epithelium (Fig. 7).

The simulation considered the tear film, epithelium and stroma. For the porcine cornea samples, as well as the human cornea with a thin epithelium, the inserted thickness of the layers was based on the immunohistological results (Figs. 2 and 5). For the human cornea with intact epithelium, the values were taken from the existing literature^14,16^.

Wavelength dependence of the penetration depth was observed in the porcine epithelium, with slight increases between 200 and 270 nm (Fig. 7a). In the human cornea with a non-intact epithelium, penetration increased notably for wavelengths > 230 nm, while in corneas with intact epithelium, the increase was less pronounced (Fig. 7b, c). These differences were attributed to the varying optical properties of the epithelium and stroma and the thickness of the epithelium.

While the absorption coefficient in the epithelium remained stable across wavelengths, the stroma’s absorption coefficient increased significantly for wavelengths > 240 nm. The absence or thinning of the epithelium in human corneas increases the influence of the stroma, which is characterized by a low absorption, resulting in a deeper light penetration.

As illustrated in Fig. 7, penetration depth correlates with epithelium thickness. In porcine corneas (epithelium of approximately 110 ± 2 μm), photons with wavelengths < 240 nm primarily reach the superficial layers of the epithelium, without reaching its basal layer (Fig. 7a, d, g). In human corneas with a non-intact epithelium (approximately 26 ± 2 μm), 10% of the intensity of the far UV-C light (< 240 nm) reaches the anterior stroma (Fig. 7b, e, h).

For the conventional UV-C radiation at 254 nm, the simulation of the human cornea with non-intact epithelium, indicates deep light penetration, reaching the posterior stroma (Fig. 7k).

Simulation of the light into healthy human corneas, considering an intact, 50 μm thick epithelium (Fig. 7c), showed that 10% of the intensity of the wavelengths < 240 nm reaches the basal epithelium (Fig. 7f, i). Thus, the light penetration depth of the intact human cornea is lower than that of the human cornea with a non-intact epithelium, but higher than that of the porcine cornea.

## Discussion

Far UV-C radiation has been widely studied for its germicidal properties and proposed for use in environments where humans are present^9,11,24–28^. However, before safe implementation, especially in scenarios involving potential eye and skin exposure, its biological effects must be carefully assessed. Direct irradiation and sampling of in vivo human eyes is neither ethical nor practical; therefore, the present study provides a comprehensive safety assessment of far UV-C radiation on the cornea, using in vitro, ex vivo models combined with light simulation. Three different models were evaluated: RHCE, ex vivo human corneas and porcine corneas. Each offers distinct advantages and limitations.

First, DNA damage was analyzed in vitro in RHCE immediately and 24 h after single and multiple exposures. RHCE was selected because it remains vital until the time point of use, allowing the study of multiple irradiations and delayed analysis. However, strong damage was observed even at low doses, especially at wavelengths > 290 nm. Furthermore, no effective DNA repair was observed, in contradiction to previous investigations^21,22^. Similar effects were observed using reconstructed human mucosa models, highlighting that repair function depends strongly on the cells used^26^. This indicates that the epithelium alone is not sufficient to analyze the UV-induced damage and full corneas are necessary.

The availability of fresh, intact human cornea remains challenging. Cornea samples from cadaveric donors are rarely available in a fresh condition. Cornea samples from transplantation banks offered a good model for studying the full cornea structure; however, detailed examinations showed that epithelial thickness often does not represent the intact epithelium of the native eye. After transplantation, the epithelium regenerates rapidly, so the reduced thickness does not affect transplantability. However, it may have an impact on our studies, since epithelial thickness affects light penetration and subsequent DNA damage. Endothelial detachment also occurred in some samples during the experiments.

To address these limitations, ex vivo porcine corneas were included as a supplementary, exploratory model. The structure of the porcine cornea is comparable to that of the human cornea^22 ^ but the epithelium is thicker than human cornea^29,30^. These samples were used to determine the optical properties of the cornea, and to compare simulated UV penetration profiles with observed UV-induced DNA damage for porcine and human cornea. The penetration depth of the light is crucial to explain the damage as shown in skin safety investigations^11^.

In the skin, the stratum corneum–the outermost layer–effectively absorbs and reflects far UV-C radiation, thereby reducing its penetration into the deeper layers of the skin and protecting germinative cells in the basal layer from UV-induced DNA damage. Previous studies simulating penetration depths between 200 and 300 nm have shown a wavelength dependence, with longer wavelengths penetrating deeper, whereas far UV-C reach only the superficial layers of the skin^11^. In contrast, the cornea, which lacks a stratum corneum, allows deeper far UV-C penetration^1,11^. Although the eye is more vulnerable to UV damage than the skin, it has higher levels of genotoxic stress response proteins like DNA damage-binding protein 2, Xeroderma pigmentosum group C protein, and tumor suppressor p53, enabling more efficient DNA repair and greater resistance to UV-induced damage^16^.

In the eye, the tear film and the epithelial layer of the cornea, serve as the outermost barriers and significantly reduce light penetration^7,16^. However, consistent with previous studies, the present study suggests that the tear film alone did not fully block light far UV-C, evidenced by the observed DNA damage in human and porcine cornea despite the presence of natural human tears during irradiation or the inclusion in the simulation of penetration depth. This is likely due to the limited thickness of the tear film (5 μm)^16,31,32^.

The corneal epithelium, is highly effective at absorbing in the UV-C range, due to its high concentration of ascorbic acid and proteins such as tryptophan^33,34^. In the present study, the absorption and reflection coefficients of the corneal epithelium increased significantly at wavelengths < 240 nm, thereby reducing penetration into deeper ocular structures^7,34–36^. Thus, the thickness of the epithelium seems to be a key factor in UV light penetration and potential damage to deeper layers. When comparing the three models—RHCE, human corneas, and porcine corneas— with different epithelial thicknesses, it was observed that the human corneal samples (26 ± 2 μm mean epithelium thickness) exhibited a deeper UV penetration depth, accompanied by a more pronounced damage. In contrast, models with a thicker epithelium, the RHCE (79 ± 2 μm) and porcine corneal epithelium (110 ± 2 μm), demonstrated reduced damage after UV irradiation.

Due to limited availability of fresh human corneal samples, UV penetration depth was simulated based on the optical properties from porcine corneas. In human corneas with a normal epithelial thickness (approximately 50 μm)^14,37^ simulations show that less than 10% of the intensity of wavelengths < 240 nm reaches the anterior stroma. The integration of simulations and immunohistological analysis suggests that 30–40% intensity of far UV-C radiation is needed to induce CPD-type DNA damage. Therefore, exposure at 233 nm is expected to cause damage to the whole epithelium, while 222 nm irradiation impacts only half of the epithelium with little to no involvement of the basal cells. This aligns with previous in vivo human studies, where 222 nm irradiation up to 75 mJ/cm^2^ caused no acute or chronic ocular damage, nor changes in visual acuity, refractive error, or corneal endothelial cell density, though temporary discomfort was reported^31,38,39^.

Some discrepancies with human corneas are expected, as simulations used porcine cornea optical parameters. As stated above, the porcine cornea has been shown to have a morphology similar to that of the human eye, however the thickness of its layers is known to be thicker than in the human eye^29,30^. Additionally, differences in chromophore composition, cell structure, and organization influence UV transmission through ocular tissues^40^. Notably, for 254 nm, the simulated penetration depth did not align with histology results from human corneal samples, likely due to the thin or detached epithelium in some of the samples, which allowed for deeper UV penetration. Additionally, the human samples were cultured for several days between collection and experimentation, whereas the porcine samples were used freshly. As prolonged storage has been shown to increase light penetration, this storage effect likely contributed to the observed differences^41^. Additionally, the freezing process inherent to the methodology may have influenced the results.

Overall, immunohistochemical analysis and optical properties analysis in the present study demonstrated a clear wavelength dependency of penetration depth into the cornea, consistent with previous skin studies^11,21,28^. UV-B radiation at 3 mJ/cm^2^ and conventional UV-C radiation at 254 nm 40 mJ/cm^2^ showed in human cornea samples with a thin epithelium deep penetration and higher DNA damage, even affecting stromal layers and endothelial layers, respectively. Such finding supports previous reports linking conventional UV-C to the onset of ocular pathologies^40,42^. Although the UV-B dose does not exceed 10% of one minimal erythema dose for skin types II–III, it is important to note that the eye has been shown to receive only 5–6% of this dose due to anatomical features that provide protection.

In contrast, far UV-C wavelengths (222 nm and 233 nm) showed significantly reduced penetration, as demonstrated in previous skin studies^11,43,44^. Irradiation with 222 nm at 40 mJ/cm^2^ in human corneas induced less DNA damage to superficial epithelial cells. Irradiation with 233 nm at 40 mJ/cm^2^ induced more damage to epithelial cells than 222 nm, but remained superficial without affecting deeper stromal or endothelial layers.

Comparing our investigations with other studies, similar results were obtained. Recent studies in 3D models similar to those used in the present study showed that wavelengths between 215 and 235 nm also induced about 20% of dimers in the most superficial layers of the epithelium without stopping proliferation of germinative cells^45^. Kaidzu *et al.* evaluated the ocular safety using porcine and rat corneal limbus and reported superficial epithelial cell damage after irradiation with 222 nm at 1500 mJ/cm^2^, with no photokeratosis even at 3500 mJ/cm^222^. Similarly, 235 nm irradiation at 30 mJ/cm^2^ penetrated about half the epithelium^22^. Nevertheless, Kaidzu *et al.* conducted the studies on anesthetized in vivo rats, where eye closure affected exposure, and on ex vivo porcine corneas, which have a thicker epithelium (110 μm) compared to humans (50 μm)^14,37^. Although these models are valuable, they do not perfectly replicate human ocular conditions. Species-specific differences in epithelial thickness (as noted above) influence UV penetration and the induction of DNA damage^40^.

Besides the damage itself, the DNA repair is critical for safety assessment under repeated exposures. In the present study, DNA damage repair was detected 24 h after exposure across all human cornea samples. Only the 254 nm group showed increased DNA damage in the stroma and the endothelium after 24 h. This increase may be due to the photosensitizing effects of chromophores within the samples^1^ and potential variations among donors. The repair capacity was dependent on the corneal layer, with lower repair in the stroma and the endothelium compared to the epithelium. This is due to the absence of a total natural renewal cycle and regenerative processes in deeper layers^40,46^. Superficial epithelial cells naturally turnover within 24 h and renew weekly (7–10 day lifespan)^16,46^. Thus, while far UV-C irradiation causes damage to the corneal epithelium, its robust repair capacity and rapid turnover minimize long-term risks^7^.

However, RHCE exhibited less efficient repair and showed DNA damage accumulation after multiple exposures. This raises concerns about repair efficiency under repeated far UV-C exposure and highlights potential limitations of far UV-C radiation for decontamination in public spaces when multiple exposures are necessary. Nevertheless, further research is needed to determine if this effect is due to the in vitro model’s limitations in DNA damage repair and in the complex cell turnover cycle.

In conclusion, the results have shown that direct irradiation at 233 nm could raise more ocular safety concerns for room disinfection due to potential involvement of basal epithelial cells than far UV-C at 222 nm which only induce superficial epithelial damage. Reduced exposure in real-world settings (approximately 5.8% of the maximum dose) due to anatomical protection, blinking, and absorbing or reflecting surfaces^47 ^along with an intact corneal epithelium, may limit damage to superficial epithelial layers. This damage can be repaired by epithelial turnover, preventing stromal and endothelial exposure and ensuring no long-term harm. Nevertheless, studies under controlled conditions with multiple exposures are needed.

## Materials and methods

### Reconstructed human cornea epithelium (RHCE)

The first studies of UV-induced DNA damage were carried out using RHCE. RHCE were used to study both the immediate and 24 h post-irradiation effects, as well as the effects of repeated exposures. The EpiCorneal ocular tissue (COR-100) models (MatTek Corporation, Ashland, MA, USA) consisted of normal human corneal epithelial cells. The RHCE was cultivated at 37 °C, 5% CO_2_ and 100% humidity in 6-well plates containing 1.0 mL of COR-100-ASY maintenance medium (MatTek Corporation, Ashland, MA, USA), which was pre-warmed to room temperature for 30 min. Prior to irradiation, the tissue models were rinsed and transferred into PBS, where they remained throughout the irradiation period (Table 1), to prevent desiccation and to avoid potential interactions between UV light and cell culture medium. Tissues were removed from the incubator only during the irradiation process. During this period, temperature was monitored and maintained at 20 °C. Samples that were not fixed immediately after irradiation were recultivated in COR-100-ASY medium for 24 h before fixation. To assess DNA damage after multiple exposures, the models were irradiated four times with two hour breaks in between, followed by 24 h re-cultivation and fixation.

### Excised human cornea

Ex vivo human cornea samples were used as a second model to investigate DNA damage in all corneal layers and its penetration depth after UV irradiation. This approach was chosen to evaluate all layers of the human cornea including not only the epithelium but also the stroma and endothelium.

The samples (that were officially not suitable for corneal transplantation) were obtained from the Corneabank of the Clinic and Polyclinic for Ophthalmology at Medical University of Rostock, Rostock, Germany (A 2020 − 0108). Informed consent was obtained from all subjects. All studies were undertaken with the approval of the Ethics Committee of the Charité – Universitätsmedizin Berlin (EA1/324/19) and were conducted according to the declaration of Helsinki. The cornea samples were obtained from donors and immediately placed in the culture medium I (PAN Biotech GmbH, Aidenbach, Germany) and stored at room temperature. Two to five days before the experiment, the corneas were transferred to culture medium II (PAN Biotech GmbH, Aidenbach, Germany) supplemented with dextran as a dehydrating agent to reverse corneal swelling. The cornea samples were transported to the laboratory and prepared for UV irradiation. During this period, temperature was monitored and maintained at 20 °C. Prior to irradiation, they were cut into four pieces and rinsed several times with PBS solution (Gibco™, Thermo Scientific Inc., New York, USA) to prevent any photosensitivity of the culture medium. The irradiation protocol is described in Table 1. To prevent desiccation, the samples were placed on wet paper humidified with 5 µL of human tears prior to irradiation. Human tears were collected from a single healthy donor (natural crying) and pipetted (5 µL) to the corneal surface every 30 s during irradiation (Table 1) to prevent desiccation and simulate in vivo tear film conditions. Artificial tears were evaluated for their spectral absorption characteristics (see Supplementary Fig. 3) but were not used in the irradiation experiments due to their higher absorbance in the far UV-C range.

For DNA damage analysis, the samples were either fixed in formalin immediately after irradiation or subjected in culture medium II (PAN Biotech GmbH, Aidenbach, Germany) for 24 h at 37 °C in a 5% CO_2_ environment prior to fixation.

### Excised Porcine cornea

Due to the thin epithelium or absence endothelium in human corneal samples, two porcine corneas were included in a supplementary experiment to qualitatively assess UV penetration in the cornea with intact epithelial and stromal layers. These samples were used to qualitatively compare simulated penetration depth with observed DNA damage. Due to the exploratory nature and limited sample availability, no statistical or power analysis was conducted.

The porcine eyes were provided by a local butcher (from animals slaughtered as part of routine food production), which was approved by the Veterinary and Food Inspection Office, Dahme Spreewald, Germany. Porcine heads were obtained from slaughterhouses on the day of experimentation and transported to the laboratory on ice to prevent tissue degeneration. Directly after receipt, the eyes were separated from the head and excess tissue was removed. During this period, temperature was monitored and maintained at 20 °C. The inner portions were removed until only the cornea remained, the tissue was rinsed several times with PBS and divided into four sections prior to the radiation experiment. The same irradiation protocol used for human corneas was applied and is described in detail in Table 1.

To prevent desiccation, porcine corneas were placed on paper moistened with 5 µL of human tears, with additional tears (5 µL) applied to the surface every 30 s during irradiation. Due to the limited availability of porcine tear fluid, human tears were used to simulate natural tear film conditions and to maintain consistency with the protocol used for human corneas.

### UV radiation

The type and dose of radiation varied among the different corneal models. Table 1 summarizes the specific conditions used in each experiment.

Initially, RHCE were irradiated with 222 nm and 233 nm at doses previously demonstrated to have bactericidal activity. However, when applied to human cornea samples, these doses caused nearly complete DNA damage in all epithelial cells (data not shown). As a result, for human the dose for 233 nm was reduced to the minimum effective bactericidal dose, and this adjusted dose was subsequently used for all radiation types (233 nm, 222 nm, and 254 nm) to eliminate dose-dependent effects and ensure comparability across conditions. The irradiation doses for the porcine cornea were equivalent to those used in the ex vivo human cornea irradiation experiments to enable direct comparison.

**Table 1:** Overview of radiation sources, applied doses and exposure time in the irradiation of different corneal models. RHCE - reconstructed human cornea epithelium.

Corneal models	In vitro RHCE	Ex vivo human cornea samples	Ex vivo porcine cornea samples
DNA damage analysis	Immediately and 24 h after exposure	Directly and 24 h after exposure	Directly after exposure
Dosing protocols	Single and multiple exposures*	Single exposures	Single exposures
Wavelength	Dose in mJ/cm^2^ (exposure time in min: sec)		
222 nm	150 (10:25)	40 (02:47)	–
233 nm	60 (08:20)	40 (05:33)	40 (05:33)
254 nm	–	40 (01:15)	40 (01:15)
UV-B (280–400 nm)	3 (01:26)	3 (01:26)	3 (01:26)

*RHCE was also exposed to multiple irradiations, and DNA damage was analyzed after 24 h.

**Fig. 8:**
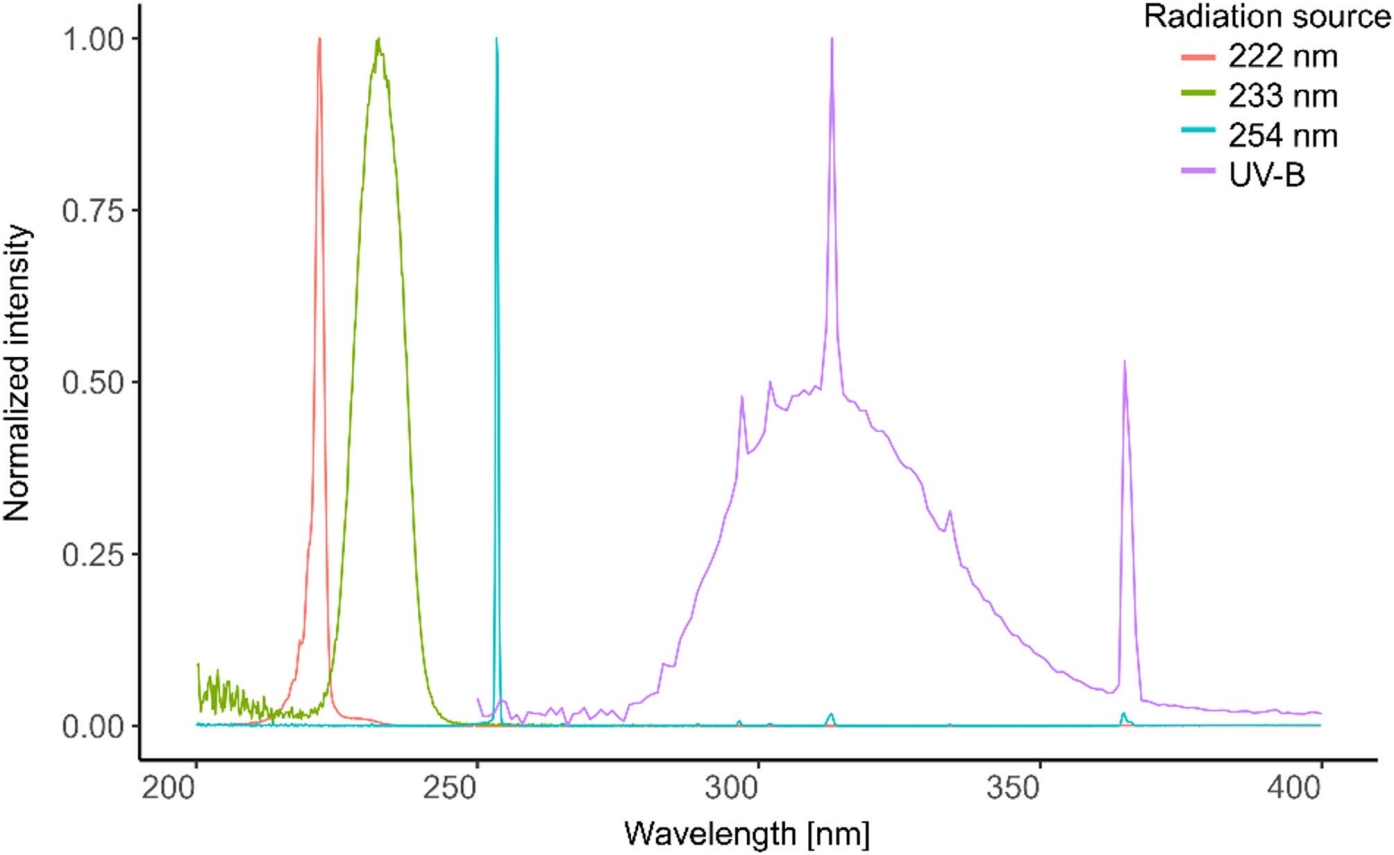
Emission spectra of applied UV light sources. The normalized spectra are shown for 222 nm (red), 233 nm (green), 254 nm (cyan) and broadband UV-B (violet)^26^.

Conventional UV-C radiation was used with a wavelength of 254 nm (0.54 mW/cm^2^, mercury gas discharge lamp, sglux GmbH, Berlin, Germany). Additionally, the well-established 222 nm Kr-Cl excimer lamp (3.34 mW/cm^2^, ExciJet222 30–130 Kit (111073), USHIO Deutschland GmbH, Steinhöring, Germany), with a short pass filter to suppress wavelengths > 230 nm, and a 233 nm far UV-C LED source with 12 nm FWHM LEDs (0.041 mW/cm^2^, Ferdinand-Braun-Institut gGmbH, Berlin, Germany) with a short-pass optical filter with a cut-off wavelength of > 240 nm were investigated. For risk assessment, a broadband UV-B lamp (41 µW/cm^2^, TH-1E from Cosmedico^®^, JW Sales GmbH, Stuttgart, Germany) containing similar fractions of UV-B (280–315 nm; 50.69%) and UV-A (315–400 nm; 48.21%) at a dose of 3 mJ/cm^2^ was also used in this study (Fig. 8). The inclusion of broadband UV lamp was used to simulate solar irradiation and the selected UV-B dose does not exceed 10% of one minimal erythema dose for skin types II–III, which is equivalent to just 3–6 min of sun exposure — an amount that is likely unavoidable for skin in daily life^28^. Non-irradiated cornea samples were included as negative controls. These samples underwent the same handling procedures as the irradiated samples; additionally, they were kept outside the incubator for 2 minutes to simulate the irradiation period, but without UV exposure. They were then either fixed immediately or recultivated for 24 h before fixation.

The distance between the sample and the lamp was 5 cm for the 222 nm, 233 nm, and 254 nm sources, and 28 cm for the UV-B lamp. Irradiance of the 254 nm, 222 nm, and 233 nm UV-C sources was measured with the UV radiometer SXL55 with a SiC UV-C sensor (sglux GmbH, Berlin, Germany), and for the broadband UV-B lamp with an ILT 1400 radiometer photometer (SEL240) (International Light Technologies Inc., Peabody, MA, USA).

### Analysis of DNA damage

For DNA damage analysis, three regions of interest were considered, from superficial to deep: the epithelium, the stroma (subdivided into anterior and posterior stroma, Supplementary Fig. 2), and the endothelium. The most common premutagenic lesions were evaluated: CPD and 6-4PP. The DNA damage was evaluated directly and 24 h after irradiation by reculturing corneal samples in medium at 37 °C with 5% CO_2_, employing the immunohistochemical method previously described^48^.

The samples underwent fixation in a neutral buffered 10% formalin solution (Merck KGaA, Darmstadt, Germany) and were subsequently embedded in paraffin (Histosec™, Merck Millipore GmbH, Darmstadt, Germany) overnight. Paraffin blocks were prepared per sample and 1–2 μm thick sections were cut from these blocks. Sections were dewaxed and subjected to a heat-induced epitope retrieval step at pH 6 prior to immunohistochemistry.

Sections were incubated with either anti-6-4PP (clone 64 M-2, Cosmo Bio, 1:400) or anti-CPD (clone TDM-2, Cosmo Bio, 1:2,000 for human samples and 1:5,000 for porcine samples) for 30 min at room temperature. For detection, Dako REAL™ Detection System, Alkaline Phosphatase/RED, Rabbit/Mouse (Agilent Technologies) was used. Nuclei were counterstained with hematoxylin (Merck Millipore) and slides coverslipped with Kaiser´s glycerol gelatine (Merck Millipore). Negative controls were performed by omitting the primary antibody. Manual counting of positive cells in relation to all cells using an AxioImager Z1 microscope (Carl Zeiss MicroImaging, Inc., New York, USA) and a Vectra3 multispectral microscopy system (Akoya Biosciences, Marlborough, MA, USA). Analysis was conducted separately for each corneal layer, and the findings were presented as the percentage of positive cells within a given image.

For each sample, five high power fields per region were selected, and at least 100 cells per field were required for analysis. High-power fields in this study were acquired at 400× magnification, corresponding to a resolution of 0.25 μm per pixel. In the case of the endothelium, where cellularity was often lower, a minimum of 100 total cells per sample was required for inclusion.

Some human corneal samples exhibited tissue detachment—particularly of the epithelium or endothelium—due to natural variability or handling during processing. In such cases, detached areas were excluded from analysis, and only intact regions were evaluated. Samples with insufficient cell counts were excluded from the analysis.

Consequently, the number of samples included per irradiation group varied depending on tissue integrity, as only regions meeting the defined quality and cell count criteria were analyzed.

### Optical properties of the eye and penetration depth

Given the limitations in thickness or condition of the epithelium of the various corneal models, the optical parameters between 200 and 300 nm wavelengths of the porcine eye were measured to simulate light penetration and then compared with the immunohistological sections. The resulting data were then extrapolated to the human cornea to estimate potential damage in an intact human eye.

Biopsy samples, 8 mm in diameter, were extracted from porcine corneas, flattened, and frozen at − 20 °C on the day of slaughter. They were processed within 14 days. Following embedding in cryomedium (Tissue Freezing Medium, Leica Biosystems, Richmond, IL, USA), 40 μm-thick horizontal sections were cut using a cryotome (Microm Cryo-Star HM 560, MICROM International GmbH, Walldorf, Germany). Confocal laser scanning microscopy (CLSM) (LSM 700, Carl Zeiss AG, Oberkochen, Germany) with a 10× objective was used to image biopsy sections. Initially, 10× images were taken at the epithelial-stromal interface to distinguish their morphologies. Subsequently, tile scans of entire sections were performed to map layer locations relative to final measurement areas and identify any tissue holes (Supplementary Fig. 4).

Epithelial and stromal thin Sect. (40 μm) were analyzed for total transmittance and total reflectance between 200 nm and 300 nm with a spectrophotometer (Lambda 650™, PerkinElmer LAS GmbH, Rodgau, Germany) using an integrating sphere. The spectral bandwidth of the spectrophotometer was 5 nm. A detailed description of the procedure can be found in Zamudio Díaz *et al*.^11^.

Optical parameters, specifically the absorption µ_a_ and effective scattering coefficients µ_s_’, were determined using iMCS, as outlined by Meinke *et al.*^49^. This method employs iterative forward Monte Carlo simulations to estimate the optical parameters µ_a_ and µ_s_’, utilizing the Henyey–Greenstein phase function along with experimentally obtained reflection and transmission values (RtM and TtM). The refractive index of the cornea was taken from Fig. 3a of the publication by Inniger *et al.*^41^. A linear extrapolation of the refractive index was carried out for the wavelength between 250 and 300 nm, which is based on the course in water.

A forward Monte Carlo ray tracing simulation model was developed for the porcine cornea using wavelength-dependent material properties derived from µ_a_ and µ_s_’ obtained from iMCS. The model incorporated the mirror boundary condition and the absorption characteristics of real human tears (Supplementary Fig. 3). The porcine cornea model comprised a tear film (5 μm), epithelium (110 μm), and stroma extending infinitely, based on histological sections.

Using the same optical properties as those of the porcine cornea, simulations were also conducted to estimate light penetration depth and distribution within the human cornea, both with and without an intact epithelium. In the model of the human cornea with non-intact (thin) epithelium, the structure comprised a 5 μm tear film, a 26 μm epithelium (based on histological sections), and an infinitely thick stroma, while the intact human cornea model included a 5 μm tear film, a 50 μm epithelium, and an infinitely thick stroma^14,16^.

### Statistical analysis

Statistical analyses were conducted using IBM SPSS^®^ Statistics version 26 (IBM, Armonk, NY, USA). For datasets involving multiple measurements, the outliers, the mean and standard error of the mean (MW ± SEM) were calculated. In the text, values presented in this format refer to the mean ± SEM. Data normality was assessed using the Shapiro–Wilk test. For comparison on DNA damage, non-parametric methods were employed. The Kruskal-Wallis ANOVA was used to evaluate differences across multiple groups, followed by Bonferroni-corrected post hoc tests to identify pairwise group differences. A p-value < 0.05 was defined statistical significance. Data visualization was performed using OriginPro^®^ version 2019b (OriginLab Corporation, Northampton, MA, USA).

## Electronic supplementary material

Below is the link to the electronic supplementary material.

Supplementary Material 1

## Data Availability

Data that support the findings of this study are available from the corresponding author Daniela F. Zamudio Díaz (d.zamudio.diaz@campus.tu-berlin.de) upon reasonable request.
