# Supplementary material for: Corneal safety assessment of germicidal far UV-C radiation
Source: Sci Rep. 2025 Jul 5;15:24052. doi: 10.1038/s41598-025-09241-2 (PMC12228727; doi:10.1038/s41598-025-09241-2)
Supplement: Supplementary file 1 — Supplementary Material 1 [file 41598_2025_9241_MOESM1_ESM.docx]

Supplementary Information

## **Results**

## **Depth of** **DNA damage in human cornea and porcine cornea**


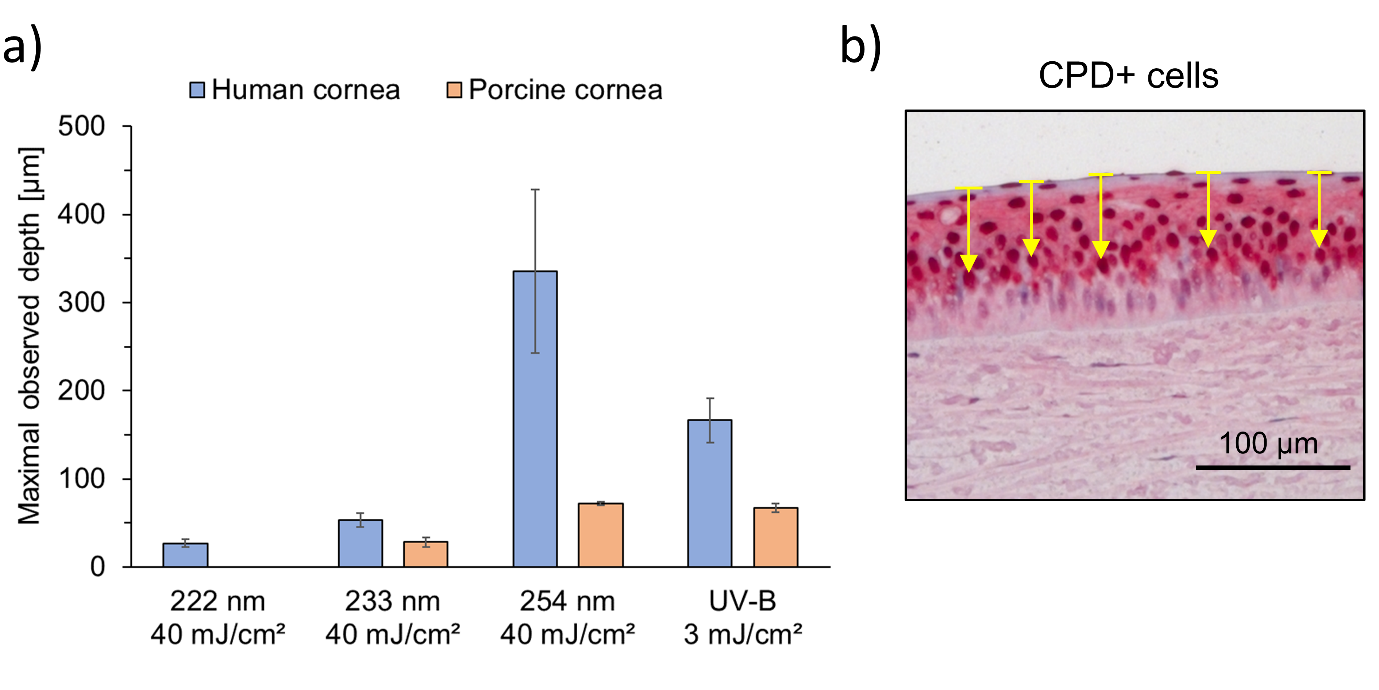


Figure S1: Depth of DNA damage in human (blue) and porcine (orange) corneas (a), quantified by measuring the distance from the corneal surface to the deepest cyclobutane pyrimidine dimer (CPD)-positive cells observed in five regions per tissue section. The samples were irradiated with 233 nm, 254 nm at 40 mJ/cm² and broadband UV-B (280–400 nm) at 3 mJ/cm². Human corneas were additionally irradiated with 222 nm at 40 mJ/cm². The data show mean ± SEM. n = 2–10. Representative immunohistochemically stained section of porcine cornea irradiated with 254 nm at 40 mJ/cm², illustrating how the penetration depth of CPD-positive cells was assessed (b). CPD positive cells are stained in dark red.

## **Analysis of DNA damage in upper and lower stroma**

For DNA damage analysis, 5 high power fields were chosen and the positive cells in relation to all cells were counted. The most common premutagenic lesions were evaluated: CPD and 6-4PP. The stroma was subdivided into anterior and posterior stroma (Figure S1).


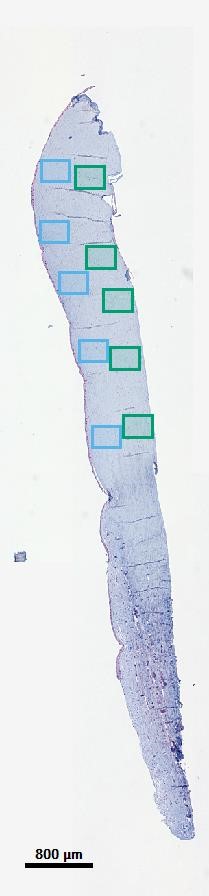


Figure S2: Representative immunohistological analysis of DNA damage in the stroma. Due to its thickness, the stroma was divided into anterior (blue) and posterior (green) regions. Five high-power fields per region were selected, and the number of positive cells was counted in relation to the total number of cells.

## **UV absorption of human and artificial tears**

To assess the potential protective effects of tears against UV radiation, the absorbance properties of two tear samples were examined: real human tears and artificial tears (Section 2.1). Initially, a concentration of 100 µL/mL was used, which reached a saturation point at wavelengths below 225 nm, necessitating a reduction in concentration to 10 µL/mL.

Figure S3 highlights a notable difference in absorption behavior, showing that artificial tears exhibit higher absorption levels compared to human tears. Specifically, Figure S3 indicates that the absorption of human tears increases exponentially for wavelengths below 240 nm, with a peak absorption occurring between 200 nm and 210 nm. At the wavelength of interest for this study, 233 nm, at a concentration of 10 µL/mL, artificial tears exhibited an absorption of approximately 0.20 arb. units, while human tears demonstrated an absorption of 0.07 arb. units.

To closely simulate in vivo conditions, irradiation experiments of human and porcine corneas were conducted using human tears. The UV absorption spectra of real human tears were included in the simulations of light penetration depth and distribution.


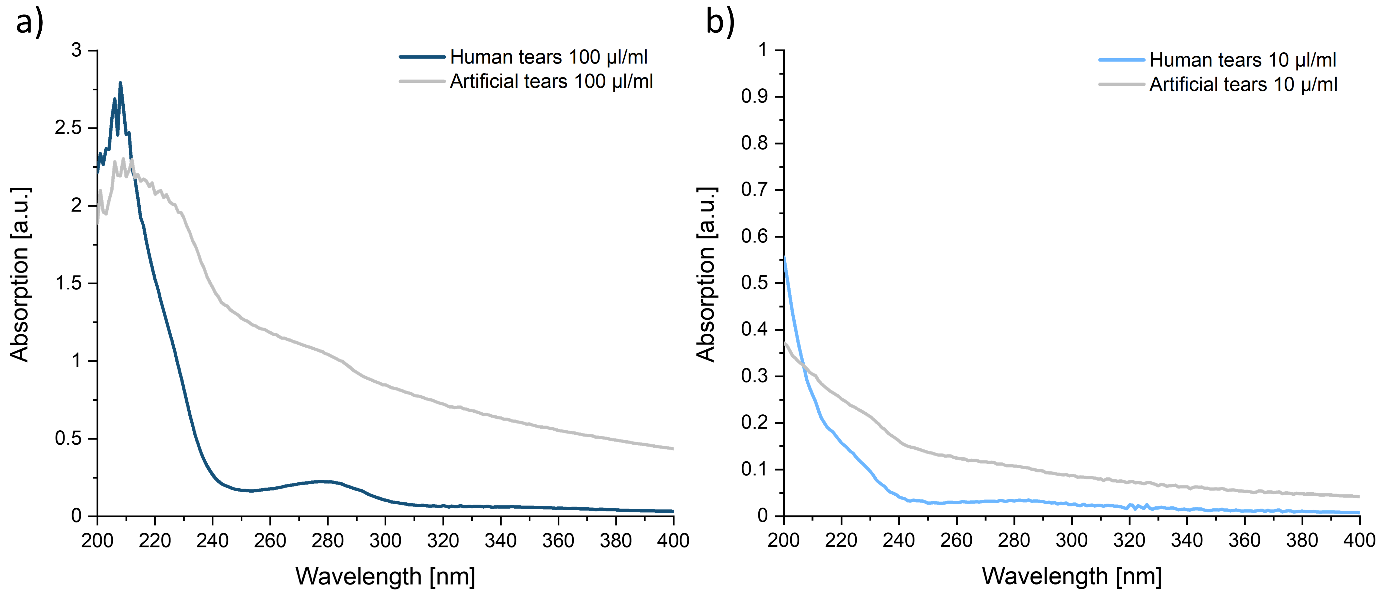


Figure S3: UV absorption of real human tears and artificial tears measured between 200 and 400 nm at two different concentrations: 100 µL/mL (a) and 10 µL/mL (b). Measurements were performed using a UV/VIS spectrophotometer. Human tears were collected from a single healthy donor. Artificial tears (Cationorm®) are a commercially available product. Samples were diluted in phosphate-buffered saline (PBS) for analysis. n = 1 for each type of tears and concentration.

## **Analysis of thin section using microscopic methods**


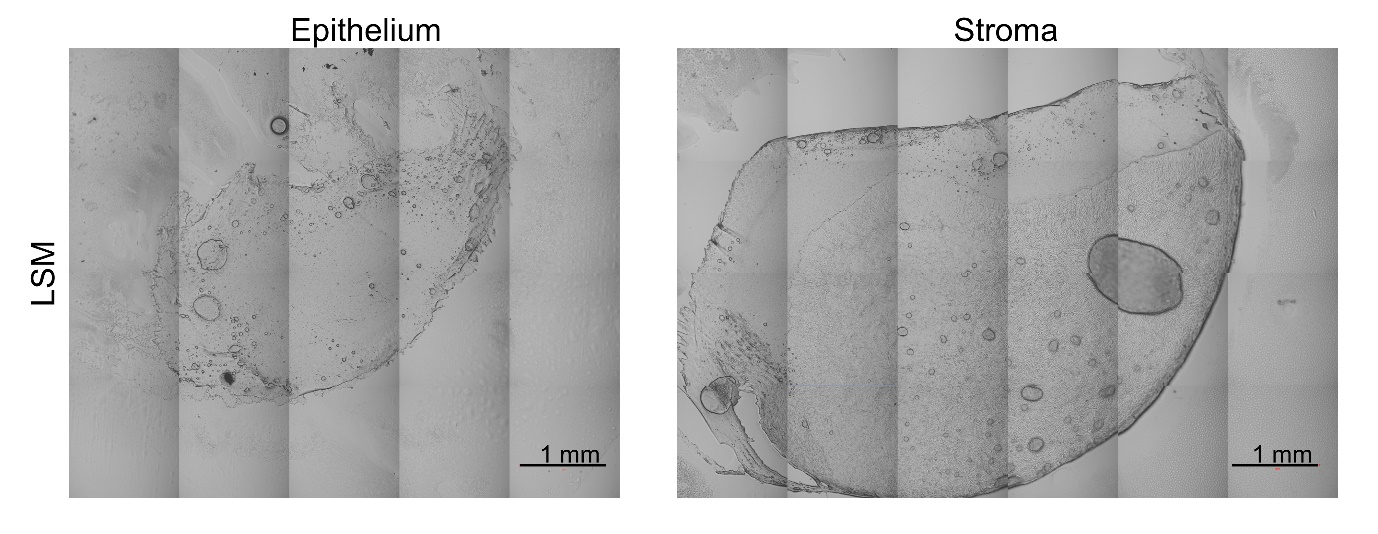


Figure S4: Example of visualization of epithelium and stroma of porcine cornea samples. Biopsies of porcine corneas were collected and embedded in cryomedium, followed by horizontal sectioning at a thickness of 40 μm. The integrity of these sections was evaluated to assess their suitability for determining optical properties. A tile scan of the entire thin section was acquired using laser scanning microscopy (LSM) with a 10× objective.

## **Materials and methods**

## **UV absorption of human and artificial tears**

To assess the potential protective role of tears against UV radiation, the UV absorption spectra of real human tears and artificial tears were measured.

Human tears were collected from a single healthy donor via natural tear production. Tears were stored at room temperature and used within 3 days for UV/VIS spectrophotometric analysis. Artificial tears were obtained from Cationorm*®* (Santen, Evry, France), a commercially available product composed of kerosene, glycerol, tyloxapol, poloxamer 188, trometamol hydrochloride, trometamol, cetalkonium chloride, and water for injection.

The spectral range examined was from 200 to 800 nm, utilizing a Lambda 650 S UV/VIS spectrometer (PerkinElmer GmbH, Rodgau, Germany). The spectral bandwidth of the spectrophotometer was 5 nm. Samples were prepared by diluting the tear solutions in phosphate-buffered saline (PBS) to concentrations of 100 µL/mL and 10 µL/mL. Subsequently, 250 µL of each solution was transferred into ultramicro cuvettes with a 10 mm path length (Hellma™, Fisher Scientific GmbH, Schwerte, Germany). The absorbance of these solutions was then recorded. For UV/VIS spectrometric analysis, background correction was performed with the cuvette together with the solvent.
